# The prototype HIV-1 maturation inhibitor, bevirimat, binds to the CA-SP1 cleavage site in immature Gag particles

**DOI:** 10.1186/1742-4690-8-101

**Published:** 2011-12-07

**Authors:** Albert T Nguyen, Christa L Feasley, Ken W Jackson, Theodore J Nitz, Karl Salzwedel, Gillian M Air, Michael Sakalian

**Affiliations:** 1 Departments of Microbiology and Immunology, University of Oklahoma Health Sciences Center, Oklahoma City, Oklahoma 73104; 2 Biochemistry and Molecular Biology, University of Oklahoma Health Sciences Center, Oklahoma City, Oklahoma 73104; 3 Warren Medical Research Institute, University of Oklahoma Health Sciences Center, Oklahoma City, Oklahoma 73104; 4 Panacos Pharmaceuticals, Watertown, MA 02472; 5 Division of Cell Biology and Biophysics, NIH/NIGMS, 45 Center Drive, Bethesda, MD 20892-6200

## Abstract

**Background:**

Bevirimat, the prototype Human Immunodeficiency Virus type 1 (HIV-1) maturation inhibitor, is highly potent in cell culture and efficacious in HIV-1 infected patients. In contrast to inhibitors that target the active site of the viral protease, bevirimat specifically inhibits a single cleavage event, the final processing step for the Gag precursor where p25 (CA-SP1) is cleaved to p24 (CA) and SP1.

**Results:**

In this study, photoaffinity analogs of bevirimat and mass spectrometry were employed to map the binding site of bevirimat to Gag within immature virus-like particles. Bevirimat analogs were found to crosslink to sequences overlapping, or proximal to, the CA-SP1 cleavage site, consistent with previous biochemical data on the effect of bevirimat on Gag processing and with genetic data from resistance mutations, in a region predicted by NMR and mutational studies to have α-helical character. Unexpectedly, a second region of interaction was found within the Major Homology Region (MHR). Extensive prior genetic evidence suggests that the MHR is critical for virus assembly.

**Conclusions:**

This is the first demonstration of a direct interaction between the maturation inhibitor, bevirimat, and its target, Gag. Information gained from this study sheds light on the mechanisms by which the virus develops resistance to this class of drug and may aid in the design of next-generation maturation inhibitors.

## Background

Despite the significant progress in the development of therapeutics against Human Immunodeficiency Virus type 1 (HIV-1), resistance to existing drugs is a continuing challenge for clinical management [1,2]. Therefore, new anti-HIV-1 agents are constantly needed. Bevirimat (BVM), previously called DSB [3], PA-457 [4], or YK-FH312 [5] potently inhibits HIV-1 replication in tissue culture and is efficacious in HIV-1 infected patients [4,6,7]. Bevirimat (3-*O*-(3'3'-dimethylsuccinyl)betulinic acid) was developed by activity-directed derivatization of betulinic acid, a plant-derived natural product [8]. Betulinic acid itself has only modest anti-HIV-1 activity, but the addition of a dimethylsuccinyl side chain at position 3 of betulinic acid enhanced its anti-HIV-1 activity 1000-fold [9]. Bevirimat is active against a wide variety of HIV-1 isolates, including those that are resistant to protease inhibitors [4]. Biochemical analyses of virus particles grown in the presence of bevirimat display a defect in capsid (CA)-spacer peptide 1 (SP1) processing. Electron microscopy revealed that such particles are aberrant, lacking a matured conical core and with a distinct electron dense immature morphology-like layer beneath the viral membrane [4,10].

The ability of bevirimat to affect only a single cleavage site in the Gag substrate suggests that Gag, rather than the protease enzyme, is the target of inhibition [11]. HIV-1 Gag is the main structural component of the virion [12,13]. After synthesis on free polysomes in the cytoplasm, myristylated Gag molecules are transported to the inner leaflet of the plasma membrane, where they polymerize and form budding structures that eventually bud off from the cell's surface [14]. The processes of budding and maturation are tightly linked, with activation of the protease coupled to, and potentially facilitating, release of the particle [15]. Maturation of the particle is required for infectivity and is accompanied by a dramatic morphological reorganization of the virion, from the spherical immature capsid with an electron lucent center, to the mature particle containing the central conical core [16-18]. The Gag precursor polyprotein is divided into sub-domains: Matrix(MA)-Capsid(CA)-SP1-Nucleocapsid(NC)-SP2-P6. The viral protease cleaves Gag in a specific order likely resulting from different inherent cleavage rates at each site [19] MA-CA-SP1 is first separated from NC-SP2-P6 at amino acid 377. Subsequent cleavages remove MA from CA-SP1 (C-terminal to amino acid 132), P6 from NC-SP2 (amino acid 448), and NC from SP2 (amino acid 432). The final cleavage separates SP1 from CA (amino acid 363). After maturation, the separated domains of Gag reorganize: the matrix protein (MA) remains associated with the viral membrane, possibly as a trimer [20], with the N-terminal portion interacting with the cytoplasmic tail of envelope, thereby in the context of full length Gag, allowing recruitment of envelope glycoprotein into the virion [21,22]; the capsid (CA) proteins assemble to form the conical core [23]; and the nucleocapsid (NC) protein associates with the viral genomic RNA, with which it condenses to form the nucleoprotein. The remaining portions of Gag: SP1, SP2, and p6, have no known functions within the mature virion. However, p6, as a part of the Gag precursor, interacts with Tsg101 to facilitate budding from the cell surface [24,25], and SP1 was proposed to function as a molecular switch that controls viral maturation [26].

The role for SP1 as a maturation regulator was initially proposed from studies where it was found that the rate of cleavage at the CA-SP1 junction could be modulated by changes at the SP1-NC cleavage site [27,28]. Studies to relate the structure of SP1 to its function have been hampered by the inability to observe order in this region in crystal structures [29,30]. Mutational analyses and modeling suggested that a helical conformation is important, as point mutations predicted to reduce helical character abrogate particle production [31,32]. NMR studies of a Gag fragment comprised of the C-terminal half of CA, SP1, and NC indicated that this region is in equilibrium between a random coil and a helical state [33]. Subsequent NMR analysis of peptides spanning the SP1 region confirmed the helical nature of this region, but required solvent conditions conducive to helix formation [34]. Recent cryo-electron tomography of immature particles indicated that SP1 forms a distinct hexameric lattice beneath the hexameric lattice formed by CA and that the density corresponding to SP1 can be fitted as a six-helix bundle. This model further suggested a mechanism by which SP1 acts as a molecular switch, whereby, following processing by the viral protease to liberate SP1 from CA, the capsid C-terminal domains extend out to make homodimeric contacts with neighboring hexamers [26]. This results in hexameric ring expansion and opening of the lattice in the mature core, as observed in several cryo-electron microscopy studies of mature assembled CA structures [17,23].

Extensive mutational studies have pinpointed the role of the CA-SP1 junction sequence in determining bevirimat activity. Substitution of SIV-specific residues into the CA-SP1 cleavage site of HIV-1 blocked inhibitor activity [35]. Since SIV is not susceptible to inhibition by bevirimat, these results suggested that bevirimat interacts directly with the HIV-1 sequence in the region of the CA-SP1 cleavage site. This idea is supported by the results of an extensive effort to develop resistance mutants *in vitro *to bevirimat [10]. Resistance could be conferred by changes at positions flanking the CA-SP1 cleavage site [reviewed in 11], with a change of alanine to valine in the P1' position arising most frequently, and also by polymorphisms within the QVT motif [36-38], while no changes were found in the sequence of the viral protease. These data suggested it should be relatively simple to replicate the activity of bevirimat *in vitro *with soluble recombinant Gag. However, attempts to do so failed [4], leading to the idea that the assembly state of Gag is a determinant for activity. This was confirmed by use of a cell-free assembly system, in which HIV-1 Gag has been modified to assemble into immature virus-like structures following *in vitro *translation that are susceptible to bevirimat inhibition of maturation [39].

Clearly, if the activity of bevirimat requires assembled Gag, then inhibition cannot simply result from recognition of the primary sequence of the CA-SP1 cleavage site. While it has been demonstrated that the inhibitor can be incorporated into whole particles [40], there has been no direct evidence that bevirimat interacts directly with the CA-SP1 cleavage site of Gag. In an effort to further characterize the target of bevirimat, we have used photoaffinity crosslinking to covalently link bevirimat analogs to immature assembled Gag. Mass spectrometry was then used to determine the regions of adduct formation within the protein. We report here the first direct evidence that bevirimat binds to its target HIV-1 Gag at the CA-SP1 junction. Additionally, because we used two photoaffinity analogs with different spacer arm lengths located at different positions on the triterpene backbone, it was possible to further characterize bevirimat's interaction site and its orientation relative to the target. *In vitro*-selected, bevirimat-resistant Gag mutants (H358Y, L363M, A364V, and A366V) as well as a synthetic mutant (ΔM377) were also examined for the ability and location of analog binding and the results support the proposed binding mode of bevirimat.

## Results

### Production and preparation of detergent-stripped immature HIV-1 virus-like particles

**Figure 1:**
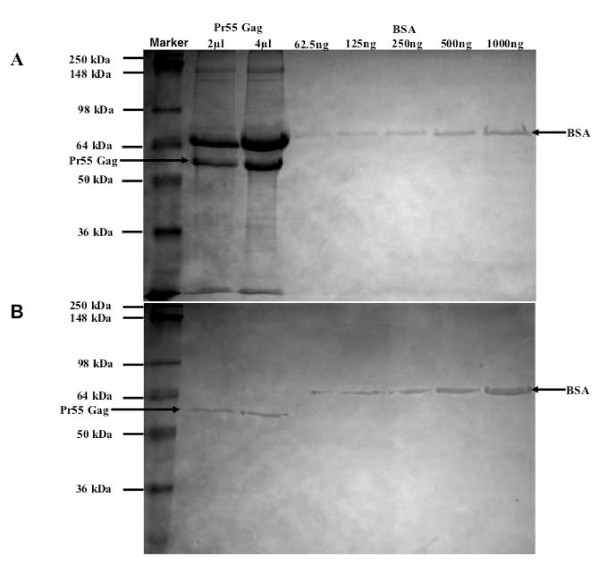
**Detergent-stripped immature HIV-1 virus-like particle purification**. (A) Coomassie blue staining of SDS-PAGE of immature VLP before detergent treatment. (B) Coomassie blue staining of SDS-PAGE of immature particles after detergent treatment. Pr55 Gag and bovine serum albumin (BSA) are indicated by the arrows. Volumes of particle sample and nanograms of BSA standards are given at the top.

Since previous analyses in cell free assays showed that bevirimat acts only in the context of assembled Gag [39], it was necessary to produce immature virus-like particles (VLP) to conduct our study. To generate sufficient material for analysis, we performed large-scale transfections of the immature particle-producing plasmid pVP-I into 293T cells. Immature particles released into the medium were filtered, purified, and collected as described. At this stage, the viral particles were heavily contaminated with fetal bovine serum (BSA) and other cellular debris as determined by Coomassie blue staining (Figure 1A). However, after stripping the membrane from the particles with mild detergent and collecting the resulting immature VLP through a discontinuous sucrose gradient, we were able to remove most of the contaminating proteins (Figure 1B). The amount of Gag in the particle preparation was quantified using a BSA standard curve run in parallel on the gel. Approximately 30 μg of HIV-1 Gag protein was used per crosslinking experiment.

### Creation of a peptide map for HIV-1 Gag after protease digestion

**Figure 2:**
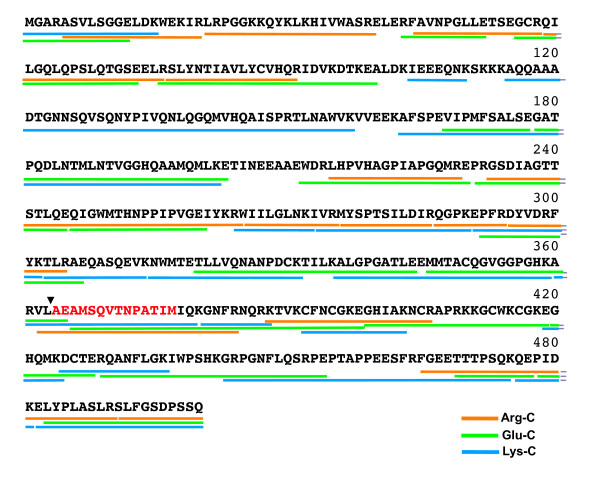
**Peptide map of HIV-1 Gag after Arg-C, Glu-C, and Lys-C protease digestions**. Only fully digested peptide fragments are shown. SP1 sequence is indicated in red. Black arrowhead points to the CA-SP1 cleavage site.

Since our aim was to identify the location of bevirimat binding site by identification of the adduct-containing peptides after protease digestion of particles, it was first necessary to perform these digestions on native Gag particles and identify the potential peptides. The resulting map of Gag peptides would then be used to identify the changes due to analog adducts and thus the binding region for bevirimat. Purified viral cores were subjected to reduction, alkylation, and protease digestion. The digestion reaction mixture was then subjected to HPLC/MALDI analysis. In order to get unambiguous identification of peptide fragments, which is critical for identifying the peptide/analog adducts, we reduced the complexity of the peptide mixture by using a shallow acetonitrile step gradient (15% to 65% in 80 minutes) and collecting small volume fractions (150 μl/fraction) from the reverse phase HPLC column. All identified peptide fragments of HIV-1 Gag after Lys-C, Glu-C and Arg-C protease digestion are shown in Figure 2. Peptide fragments covering 95.2% of the entire HIV-1 Gag protein sequence were identified. To simplify our analysis, only completely digested peptide fragments were used to construct the peptide map; partial cleavage products were excluded. Details of the mass spectra analyses of all identified peptide fragments, including signal-to-noise ratio, relative intensity, and area under the observed peak, were analyzed and recorded (Additional File 1: Additional Table 1). The removal of the first methionine and the addition of myristic acid were taken into account for mass determination of the N-terminal Gag peptide [20]. Peptide fragments smaller than 1000 daltons were difficult to identify due to the presence of the overlapping spectra of matrix ions. Peptide fragments larger than 5000 daltons were also rarely found, since large peptides were not ionized sufficiently to be detected using our MALDI-TOF instrument. Nevertheless, we were able to achieve excellent coverage and the data were complete for more than 100 amino acids each side of the region of interest, the CA-SP1 junction.

### Testing the activities of the photo-affinity bevirimat analogs

**Figure 3:**
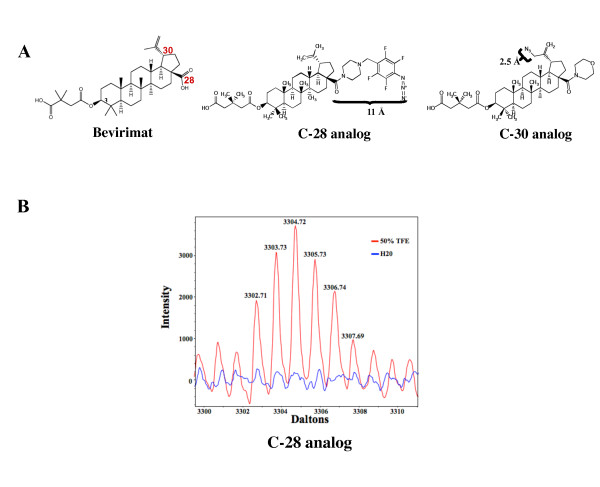
**Photoaffinity bevirimat analogs**. (A) Structures of bevirimat and the photoaffinity analogs. (B) Detail of mass spectra showing ion mass corresponding to the C-28 analog crosslinked to a synthetic peptide spanning the CA-SP1 region. Red and blue traces are spectra derived from the crosslinking reaction performed in 50% TFE or pure water, respectively. The theoretical monoisotopic mass of the synthetic peptide/C-28 analog adduct is 3302.79: monoisotopic mass of peptide + monoisotopic mass of C-28 - mass of N_2 _gas + mass of proton = 2460.29 + 869.5 - 28 + 1 = 3302.79 Da, which is in good agreement with the monoisotopic mass of the ion shown.

The structures of the two photoaffinity analogs of bevirimat used in this study are shown in Figure 3A. The C-28 analog has the azido photoreactive group attached to a tetrafluorobenzyl piperazine amide bound to carbon 28. The C-30 analog has the azide photo-reactive group attached directly to carbon 30. The IC_50 _of these analogs were determined to be similar to that of bevirimat by MT2 cell killing end point assay with virus isolate HIV-1_lllB _at 1.1 nM for the C-28 analog and 4.7 nM for the C-30 analog as compared to 1.3 nM for bevirimat. Previous work had also shown that modification of bevirimat at these positions does not significantly affect activity [8]. These results indicate that the addition of the photoaffinity groups does not appreciably alter the activity of these analogs in the whole cell assay.

To test the photo-affinity chemistry of the bevirimat analogs we first attempted to crosslink each analog to the synthetic peptide GVGGPGHKARVLAEAMSQVTNPATI spanning the CA-SP1 region. Initial experiments were performed under aqueous conditions with the synthetic peptide dissolved in either pure water or PBS. Using MALDI-TOF mass spectrometry, we were unable to detect any peptide/analog adducts. Since the CA-SP1 region may form an alpha helix [31,32], which could be critical for bevirimat binding in the context of assembled Gag, we then conducted the crosslinking experiment in 50% trifluoroethanol (TFE) in PBS. Trifluoroethanol is a nonpolar solvent that promotes helicity in peptides by desolvating the peptide backbone, thereby indirectly strengthening intra-helical hydrogen bonds [41]. Peptide/analog adducts were detected for the C-28 analog (Figure 3B) and evidence for such adducts for the C-30 analogs could be seen in the spectra, but the signal:noise ratio was low and we did not use this data. The theoretical monoisotopic mass of the synthetic peptide/C-28 analog adduct is 3302.79 Da, which matches the monoisotopic mass of the ion detected. This result not only indicated that the chemistry of the crosslinking reaction performed as expected, but also that crosslinking was more efficient when the peptide was in a helical conformation, suggesting that interaction of the analog with the peptide was greater under these conditions. Nevertheless, the relative abundance of non-crosslinked peptide in the reaction mixture was 1000 fold higher than crosslinked peptide (data not shown), which indicates the crosslinking reaction was extremely inefficient. We also observed a large proportion (~99%) of both analogs crosslinked to DMSO, which was the analog diluent (data not shown). The abundance of this side reaction could also have contributed to the low efficiency of crosslinking to the peptide under all conditions, but since bevirimat does not inhibit cleavage of unassembled Gag, the low reactivity of cross-linking is not surprising.

### Mapping bevirimat analog interaction with HIV-1 Gag within immature particles

**Figure 4:**
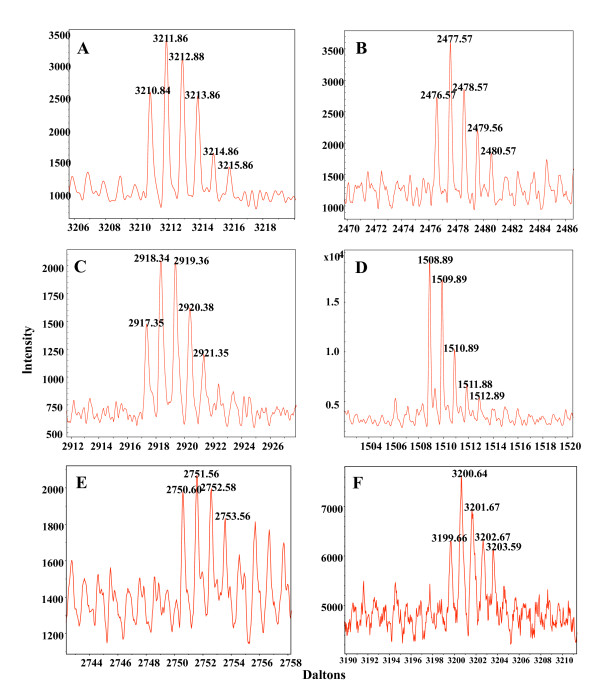
**Details of mass spectra corresponding to predicted masses for analog-peptide adducts**. (A) Lys-C peptide, Gag residues 336-359, linked to the C-28 analog. (B) Lys-C peptide, residues 291-302, linked to the C28 analog. (C) Glu-C peptide, residues 292-307, linked to the C-28 analog. (D) Arg-C peptide, residues 295-299, linked to the C-28 analog. (E) Glu-C peptide, residues 346-365, linked to the C-30 analog. (F) Arg-C peptide, residues 362-384, linked to the C-30 analog.

After confirming that both analogs possessed photoactivated crosslinking activity to at least DMSO and for the C-28 analog also to the synthetic peptide, we performed crosslinking experiments in the context of immature HIV-1 VLP. Mass spectrum details for C-28 infused and protease digested particles, incubated under either UV exposed or non-exposed conditions, were recorded and examined (Additional File 1: Additional Table 2). Following the crosslinking, the reaction products were digested with each of three proteases, Lys-C, Arg-C, and Glu-C. Experiments with the C-28 analog followed by Lys-C digestion were conducted twice. In both experiments, we observed two regions of C-28 crosslinking. Consistent with expectations given by all previous data on the bevirimat mechanism of action, we identified a peptide-analog adduct within the C-terminal region of CA just upstream from the CA-SP1 cleavage site comprising amino acid residues 336 to 359 of Gag (ALGPGATLEEMMTACQGVGGPGHK, Figure 4A). Unexpectedly, we also found a second peptide-analog adduct, within residues 291 to 302 (EPFRDYVDRFYK, Figure 4B), that lies within the Major Homology Region (MHR). The monoisotopic masses of these ions matched the theoretical monoisotopic masses of C-28/peptide adducts (Table 1).

**Table 1:** Detailed mass spectra analysis of identified crosslinked peptide fragments

Digestion/analog	Peptide Fragment	Theoretical mass + BVM(+1)	Observed mass(+1)	% difference from theoretical mass	Signal/Noise	Intensity	Area
Lys-C/C28	336-359	3210.61	3210.84	0.0072	8.1	2694	1398

Lys-C/C28	291-302	2467.30	2476.57	0.0111	8.5	2728	1318

Glu-C/C28	292-307	2917.57	2917.35	-0.0074	8.3	1480	929

Arg-C/C28	295-299	1508.81	1508.89	0.0055	26.4	19154	4786

Glu-C/C30	346-365	2750.48	2750.60	0.0044	7.2	1978	1298

Arg-C/C30	362-384	3199.79	3199.66	-0.0038	2.9	6317	5231

In contrast to the Lys-C digestion experiments, digestions with both Glu-C and Arg-C yielded crosslinked C-28 peptides corresponding to only one region of Gag. Glu-C digestion yielded only a peptide within the MHR, residues 292 to 307 (PFRDYVDRFYKTLRAE, Figure 4C). A theoretical peptide adduct including the CA-SP1 cleavage site (MMTACQGVGGPGHKARVLAE) was not observed. Likewise, Arg-C digestion of crosslinked C-28/Gag similarly yielded a peptide within the MHR, residues 295-299 (DYVDR, Figure 4D). A theoretical Arg-C peptide overlapping the region of CA found for the Lys-C digestion was also not observed, but in this case the large peptide (residues 306 to 361, molecular weight = 6036.95 Da) was likely not ionized in sufficient quantity to be detected using our MALDI-TOF instrument had it existed (Figure 2).

Since the photo-reactive group of the C-28 analog is further from the triterpene backbone (11 Å, Figure 3A) than that of the C-30 analog (2.5 Å, Figure 3A) and also oriented in a different direction from the center of the molecule, we hypothesized that the two analogs would crosslink to different locations within the Gag binding site. Crosslinking experiments with the C-30 analog followed by digestion with the three different proteases were performed as for the C-28 analog. The resulting mass spectra were also thoroughly analyzed as for the C-28 analog (Additional File 1: Additional Table 3). Two experiments with Lys-C digestion of crosslinked C-30/Gag were conducted. No peptide analog adducts were observed. However, after Glu-C digestion a peptide adduct spanning the CA-SP1 cleavage site was found corresponding to residues 346 to 365 (MMTACQGVGGPGHKARVLA, Figure 4E). The monoisotopic mass of this ion matched the theoretical C-30/peptide adduct monoisotopic mass (Table 1). Similarly, Arg-C digestion also revealed one region of crosslinking, and it too was a peptide fragment spanning CA-SP1 cleavage site, corresponding to residues 362 to 384 (VLAEAMSQVTNPATIMIQKGNFR, Figure 4F). All identified crosslinked ions had monoisotopic masses within +/- 0.015% of theoretical for the C-28 or C-30 peptide adducts (Table 1). These discrepancies from theoretical monoisotopic masses are well within the error range of our mass spectrometer, providing confidence in the data.

**Figure 5:**
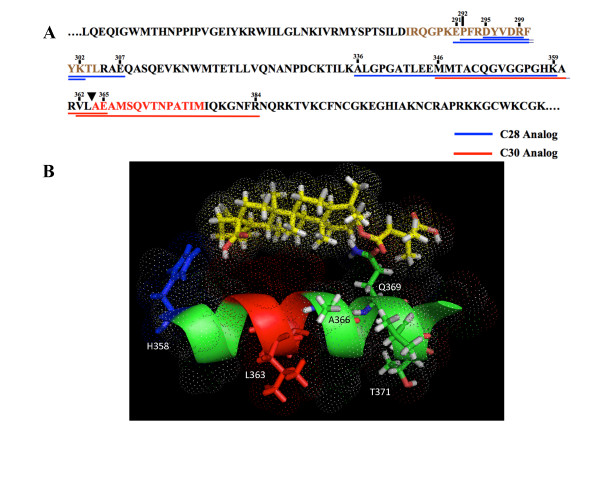
**Summary and analysis of analog-peptide adducts**. (A) Pictorial representation of all labeled Gag fragments. The SP1 sequence is indicated in red. The MHR sequence is indicated in brown. The black arrowhead indicates the cleavage site between CA and SP1. Blue and red underlines represent C-28 and C-30 analog-peptide adducts, respectively; (B) Orientation of bevirimat on its target. Bevirimat is positioned parallel to the SP1 helix. Residues H358, L363, A364, A366, Q369, A370, and T371 are indicated. The last residue of the C-28 crosslinked region is indicated in blue, the whole C-30 crosslinked region is indicated in red. The remainder of the sequence is indicated in green. The model is shown in stick mode and dots show the van der Waals surfaces.

Comparison of all identified peptide adducts allows us to infer the region within which binding of the analogs to Gag must occur (Figure 5A). Since the three different proteases used in this study produce overlapping peptides, we were able to narrow the region of analog binding based on overlap of the crosslinked peptides. The C-28 analog was crosslinked to a five amino acid residue peptide fragment (DYVDR) of the major homology region (MHR), and to a twenty-four amino acid residue peptide fragment (ALGPGATLEEMMTACQGVGGPGHK) N-terminal to the CA-SP1 junction. Most significantly, the C-30 analog was crosslinked to a four amino acid segment (VLAE) precisely spanning the CA-SP1 cleavage site.

### Lack of C-28 analog binding to the CA-SP1 region in bevirimat resistance mutants

We also conducted crosslinking experiments of the C-28 analog to four of the previously characterized bevirimat-resistant Gag mutants: H358Y, L363M, A364V, and A366V [10] and to a synthetic Gag mutant ΔM377 [39,42]. We did not observe a crosslinked peptide/C-28 analog adduct within the CA-SP1 region of these Gag mutants. However, with the exception of A366V, we did observe such a C-28 analog adduct within the MHR for all of the mutants. Details of mass spectra analyses of all non-crosslinked and crosslinked peptide fragments were also thoroughly analyzed and recorded (Additional File 1: Additional Table 4). The mass spectra data and analyses of C-28 linked to the MHR (EPFRDYVDRFYK) peptide of mutants H358Y, L363M, A364V, and ΔM377 are found in Additional File 1: Additional Figure 1 and Additional File 1: Additional Table 5.

## Discussion

The traditional approach to understanding the precise mechanism of action of a drug is to obtain an atomic resolution structure by co-crystallization of the drug bound to its target. A crystal structure of full length HIV-1 Gag is not yet available [17] and the suspected target region within Gag is disordered in the available structures of Gag fragments [29,30]. Therefore the cross-linking approach offered an alternative way to gain information on the interaction of bevirimat with its target. Photoaffinity labeling is a proven and powerful tool that has been extensively used to gain insight into drug-binding sites and mechanism of action. Using photoaffinity analogs and mass spectrometry, we were able to identify peptides that resulted from the crosslinking of the bevirimat analogs to the bevirimat binding sites within HIV-1 Gag immature VLP. Two such sites were identified, one at the CA-SP1 cleavage site and a second apparent site in the MHR. By employing different proteases and comparing the portions of overlap between the identified peptide adducts, we were able to narrow the regions of analog crosslinking. By this method we determined that the C-30 analog crosslinked within the four amino acid residues 362-365 spanning the CA-SP1 cleavage site. While only one peptide adduct was identified in this region with the C-28 analog, it occupies a site that is upstream from and proximal to the N-terminal cleavage site. Thus, we have unambiguously identified the CA-SP1 cleavage region as a target within Gag for the maturation inhibitor bevirimat. For the second binding site, located in the MHR, we were similarly able to identify a common five amino acid stretch 295-299 as the location for the adduct linkage with the C-28 analog. We did not observe a peptide adduct in the MHR for the C-30 analog.

The use of two analogs with photoaffinity groups projecting in two different directions from the inhibitor molecule allowed us to orient the bevirimat triterpene scaffold along the CA-SP1 helix. The reactive group of the C-28 analog was crosslinked N-terminal to that of the C-30 analog. In addition, the small size and restricted flexibility of the C-30 photoaffinity group constrains the positioning of the molecule such that it must be oriented with the C-28 carboxylic acid group toward CA and the dimethylsuccinyl group at C3 toward the C-terminal end of the SP1 helix (Figure 5B). The locations of the resistance mutant H358Y and the polymorphic point mutations identified by Adamson et al [43], Q369H, V370A, and Δ371T, are consistent with this orientation. It is possible for the oxygen of the C28 carboxylic acid group to make a hydrogen bond with the nitrogen on the sidechain of histidine 358. Similarly, the two oxygens of the C3 dimethylsuccinyl group could hydrogen bond with the nitrogen and oxygen on the glutamine 369 and threonine 371 sidechains, and for Valine 370 to make a critical hydrophobic contact with the 3' methyl groups of the molecule. It is plausible that changing valine to alanine would disturb an essential hydrophobic contact between the helix and bevirimat that makes V370A the most commonly observed resistant polymorphism. The existence of this critical hydrophobic interaction is supported by the fact that the dimethyl group at position 3' of the molecule enhances its antiviral activity significantly [9]. Positioning bevirimat in this way would place the molecule directly across the CA-SP1 cleavage site, providing a plausible mechanism by which binding would block access of the viral protease (Figure 5B).

**Figure 6:**
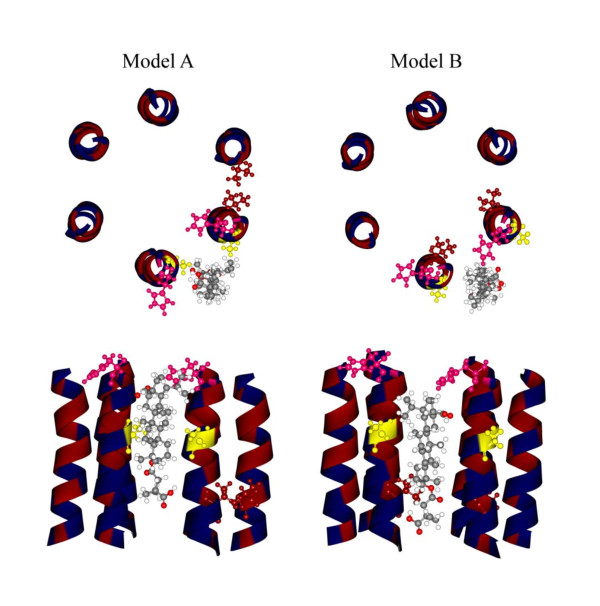
**Hypothetical models of bevirimat binding to the CA-SP1 junction**. Fitting of bevirimat into model A and B according to crosslinking and mutation data. Since the C-30 analog crosslinked within only four residues (VLAE) spanning the CA-SP1 cleavage site and the C-28 analog crosslinked within an area four residues upstream from the CA-SP1 cleavage site, we could orient the drug such that the C28 carboxylic acid group is toward the N-terminus and the C3 dimethylsuccinyl group is toward the C-terminus of the helix. Top panel: Top view of models A&B. In model A, hydrophobic and hydrophilic faces of one helix are making respective contacts with neighboring helices. In model B, the hydrophobic faces of all six helices are pointing toward the center of the bundle. Bevirimat is shown as gray ball-and-stick atoms. Interacting amino acids are also shown in ball-and-stick mode; hydrophobic residues are highlighted in red and hydrophilic residues are highlighted in blue. Models were constructed using six identical NMR structures of a peptide spanning the CA-SP1 region (PDB 1U57). Only sequence spanning the putative helix was used (GHKARVLAEAMSQVTNSATIMMQR). For simplicity, only sequence HKARVLAEAMSQVTNSAT is shown after the energy minimization process. Residue H358 is labeled in magenta. The most common *in vitro *resistant mutation, A364, is labeled in yellow. The most common resistant polymorphism, V370, is labeled in red. Bottom panel: Side view of models A&B.

NMR analyses of peptides spanning the CA-SP1 region indicates that this region can form an amphipathic α-helix in 30% TFE [34]. These results led to the interpretation of cryo-electron tomography data to show the CA-SP1 region as a six helix bundle in the ordered region of the immature Gag lattice [26]. The orientations of the helices within the bundle are unknown, but at least two plausible configurations are possible (Figure 6). In hypothetical model A, the hydrophobic and hydrophilic faces of one helix are making respective contacts with neighboring helices. Model A might be viewed as a trimer of dimers. In hypothetical model B, the hydrophobic faces of all six helices are pointing toward the center of the bundle. The existence of the helical bundle, whatever the precise configuration, is consistent with the finding from the *in vitro *HIV-1 Gag assembly system that bevirimat activity requires a higher ordered Gag structure [39]. These results support the idea that the drug's binding site might consist of more than one helix; more specifically, bevirimat might bind in the cleft between helices. This hypothesis is supported by the fact that mutations or polymorphisms that confer resistance to bevirimat lie on opposite sides of the helix (for example A364V and A366V, L363M and H358Y, Q369H and Δ371T). Interestingly, A366V not only is resistant to bevirimat, but also displays a measure of dependence in high concentration of the compound [10], further supporting the idea that this position may represent a site of direct interaction. With these mutants in mind, model A becomes more plausible as bevirimat can be positioned such that the hydrophilic and hydrophobic sides of the triterpene scaffold interact with respective sides of the helices (Figure 6 left panel, Additional File 1: Additional Table 6). This model, while hypothetical, offers a starting point for future investigation to identify residues comprising the positions of contact.

The identification of a second site for analog crosslinking in the MHR was unexpected. Two alternative interpretations are possible: 1) the MHR is a second independent binding site for bevirimat, or 2) there is only one binding site at the CA-SP1 cleavage site, but in the context of assembled Gag the MHR is in close proximity to this region such that the flexible C-28 reactive group can crosslink to it. The fact that crosslinking to the MHR was observed for most bevirimat-resistant mutants supports the idea that the MHR is a second binding site. Indeed, binding to this region, a well-characterized determinant for assembly in retroviruses [44-47], would provide a plausible mechanism for the ability of bevirimat and its precursor, betulinic acid, to inhibit particle production from cells at high concentration of compound [5,48]. Alternatively, it is possible that the MHR and CA-SP1 are in the same vicinity with two possible binding configurations for the C-28 reactive compound. The presence of bevirimat resistance mutations could alter the rotational configuration of the C-28 reactive group such that it will favor crosslinking to the MHR, but not to the peptide fragment (ALGPGATLEEMMTACQGVGGPGHK) N-terminal to the CA-SP1 junction (Additional File 1: Additional Figure 2). This idea is further supported by the fact that the C-30 analog was never found to crosslink to the MHR, which would be expected if the MHR is an independent binding site. The cryo-EM model of Wright et al. [26] brings the MHR into the vicinity of the CA-SP1 helix but the model was not precisely fitted and remains speculative.

## Conclusions

This is the first study to define the molecular target for the prototypic HIV-1 maturation inhibitor bevirimat. Although a crystal structure of the entire HIV-1 Gag is not yet available [17] and the CA-SP1 region was found to be disordered in subdomain crystallographic studies [29,30], NMR analysis of C-terminal CA-SP1-NC Gag fragment indicated that CA-SP1 is in equilibrium between random coil and helical states [33]. We propose that in the context of an immature virion, the equilibrium of the CA-SP1 region is shifted toward a helical state that would allow the formation of an ordered helical bundle. This would allow bevirimat to bind, since it requires an assembled target [39]. In the absence of bevirimat, as the viral protease cleaves MA-CA-SP1 from nucleocapsid (NC), the equilibrium may gradually shift toward a random coil. According to this model, the six helix bundle would become progressively unstructured from its C-terminal end as each helix opens to an extend conformation, which then affords the viral protease access to the scissile bond to cleave SP1 from CA. Then, as proposed by Wright et al [26], CA-SP1 cleavage may lead to expansion of the hexameric lattice of the mature particle, which would facilitate destabilization of the core to prepare the virus for uncoating following entry into the target cell. Binding of bevirimat to the CA-SP1 junction, however, likely stabilizes the six helix bundle of CA-SP1, as seen in cryoelectron microscopy analyses of bevirimat-bound budded particles [49], thereby blocking cleavage by the viral protease. Thus the critical conformational transformation in the capsid lattice is blocked, resulting in aberrant core formation and a non-infectious virus. The information gained from this study provides critical drug/target details, which in the absence of crystallographic resolution data for the CA-SP1 region of Gag, may aid the design and development of next-generation maturation inhibitors.

## Methods

### DNA constructs

pVP-I was derived from plasmid pNL4.3 by modification to contain a protease active site mutation, a deletion in *pol *and a frameshift in *env *where 'VP' stands for virus-like particles and 'I' stands for immature. When transfected into 293T cells this plasmid produces non-infectious immature VLP lacking SU and TM, and containing nonfunctional RT, IN, and PR [50]. pVP-I L363M, pVP-I A364V, pVP-I ΔM377, pVP-I A366V, and pVP-I H358Y were constructed by subcloning the 499-bp Spe-I to ApaI fragment within *gag *from pNL4.3 L363M, pNL4.3 A364V, pNL4.3 ΔM377, pNL4.3 A366V, and pNL4.3 H358Y into the pVP-I backbone. pNL4.3 mutants were constructed by site-directed mutagenesis and were kindly provided to us by Dr. Eric Freed, National Cancer Institute, National Institutes of Health. All pVP-I mutants were verified by sequencing.

### Photoaffinity analogs

The C-28 photoaffinity analog of bevirimat, PA1050097 or (3β)-28-[4-[(4-azido-2,3,5,6-tetrafluorophenyl)methyl]-1-piperazinyl]-3-(4-carboxy-3,3-dimethyl-1-oxobutoxy)lup-20(29)-en-28-one, was prepared as described in patent WO 2006053255 [51]. The C-30 photoaffinity analog, PA1070013 or (3β)-30-azido-3-(4-carboxy-3,3-dimethyl-1-oxobutoxy)-28-(4-morpholinyl)lup-20(29)-en-28-one, was prepared from (3β)-3-(4-carboxy-3,3-dimethyl-1-oxobutoxy)-28-(4-morpholinyl)lup-20(29)-en-28-one [51] by bromination at the C-30 position with *N*-bromosuccinimide in *t*-butyl methyl ether [52] followed by displacement of the resulting allylic bromide with sodium azide and tetrabutylammonium bromide in tetrahydrofuran.

### Procedure to determine IC_50 _of the analogs

HIV-1_IIIB _virus replication inhibition assays were carried out as follows: The human T-cell line, MT-2 (NIH AIDS Research and Reference Reagent Program) was maintained in complete medium (RPMI 1640 with 10% fetal bovine serum supplemented with L-Glutamine) at 5% CO_2 _and 37°C. Cells (3 × 10^4 ^per well) and an appropriate amount of virus (predetermined by titration to yield approximately 80% cell killing), were added to 96-well plates containing test compound at appropriate dilutions. After 5 days in culture, staining with XTT/PMS viability dye was used to quantify the amount of virus-mediated cell killing in each well relative to that in control wells containing no compound. Antiviral activity was measured as the percent reduction in virus-mediated cell killing. IC_50 _values correspond to the test compound concentrations calculated to result in 50% reduction in cell killing.

### Preparation of Immature VLP

293T cells were cultured in Dulbecco's modified Eagle's medium (DMEM) supplemented with 10% fetal bovine serum, 100 units/mL of penicillin, 100 μg/mL of streptomycin, and 2 mM L-glutamine, at 37°C with 5% CO_2_. 293T cells at 60%-70% confluence in 150 mm plates were transfected with 150 μg of pVP-I plasmid per plate by the calcium phosphate transfection method [53]. Cell medium was replaced with fresh medium 12 hr post-transfection. Culture supernatant was collected 48 hr later and filtered through a 0.45 μm filter. Particles were concentrated by centrifugation in a Beckman Ti 50.2 rotor at 30,000 rpm for 2 hr. The viral pellet was resuspended in 200 μl of phosphate buffered saline (PBS) and further clarified by centrifugation through a 20% w/w sucrose cushion at 41,000 rpm for 2 hr in a Beckman SW-41 rotor, and the resulting pellet was resuspended in 200 μl of PBS. Naked immature VLP were purified by treatment of resuspended particles with 1% Triton X-100 for 15-30 minutes at room temperature followed by centrifugation through a 20/44% w/w discontinuous sucrose gradient in a Beckman SW-60 Ti rotor at 60,000 rpm for 10 hr. Viral pellets were gently resuspended in PBS for at least 4 hr to overnight. The amount of Gag protein in each particle preparation was quantified using a BSA standard curve generated by ImageQuant analysis of Gag bands in an SDS-PAGE gel stained in parallel with a gel containing BSA standards.

### Photo-crosslinking of analogs to immature VLP

Each photoaffinity analog was added at a final concentration of 100 μM to 30 μg of HIV-1 Gag resuspended in 100 μl of PBS. The reaction mixture was incubated at 30°C for 2 hr with gentle shaking followed by exposure to a 312 nm UV light box (Fisher Scientific Electrophoresis System, Model FBTI81S) for 30 minutes over ice at a distance of 1 cm from the UV filter. The reaction mixture was then evaporated under vacuum to dryness in a Speed Vac Concentrator (Thermo Electron Corporation, Model SPD121P).

### Photo-crosslinking of analog to synthetic peptide

The C-28 photoaffinity analog was added to a final concentration of 100 μM in a 100 μl volume containing 1 mg/ml synthetic peptide. The reaction mixture was incubated at 30°C for 2 hr with gentle shaking followed by exposure to a 312 nm UV light box for 30 minutes over ice at a distance of 1 cm from the UV filter. The peptide/analog mixture was diluted 50 fold in 50% acetonitrile, 0.1% TFA in water and 1 μl was used for MALDI-TOF analysis.

### Reduction, Alkylation and Digestion of Analog/Gag crosslinked complexes

Completely dried crosslinking reactions were dissolved and denatured in 6 M urea with brief sonication at room temperature for 1 minute. The protein was reduced by adding dithiothreitol to a final concentration of 5 mM and incubated at 37°C for 1 hr. The alkylation reaction was carried out by adding iodoacetamide to a final concentration of 15 mM and incubated at 37°C in darkness for 1 hr with gentle shaking. Additional DTT was added to a final concentration of 15 mM and incubated at room temperature for 30 minutes to stop the alkylation reaction. The concentration of urea was reduced to 1 M by dilution with appropriate digestion buffer. Lys-C digestion buffer was 1 M urea, 25 mM Tris-HCl buffer, pH 8.5, 1 mM EDTA. Lys-C digestion was carried out at 37°C for 24 hr. Glu-C digestion buffer was 1 M urea, 25 mM ammonium carbonate buffer, pH 7.8. Glu-C digestion was carried out at 25°C for 24 hr. Arg-C digestion buffer was 1 M urea, 100 mM Tris-HCl, 10 mM CaCl_2_, 5 mM DTT, 0.5 mM EDTA, pH 7.6. Arg-C digestion was carried out at 37°C for 24 hr. A ratio of 1:30 (w/w) of enzyme/protein was used for each digestion reaction.

### HPLC separation of digestion products

Protease digestion products were evaporated to dryness under vacuum in a Speed Vac Concentrator and resuspended in 250 μl of 20% v/v acetic acid. The suspension was filtered through a 0.45 μm filter (Costar 8170, Spin-X centrifuge tube filter) in a microcentrifuge at 13,000 rpm for five minutes before loading onto a 2.1 × 150 mm C18 HPLC column (Higgins Analytical, Haisil 300 Å, 5 μM). The peptides were eluted with a 15-65% linear gradient of acetonitrile over a period of 80 minutes using a Paradigm MS4B micro-HPLC. Column effluent was monitored at 215 nm. Eighty 150 μl fractions were collected per experiment.

### Preparation of Matrix-Sample Crystals for MALDI

α-Cyano-4-hydroxycinnamic acid (CHCA) was prepared as a saturated solution (~10 mg/ml) in 50% acetonitrile and 0.1%TFA in water. 1 μl of matrix solution was applied to each sample spot on a 384 well ground steel target plate and 1 μl of peptide sample solution was added directly onto the matrix solution. Spots were dried under vacuum at room temperature. Fresh matrix solution was prepared for each run.

### MALDI-TOF Mass Spectrometry

All mass spectra were obtained on a Bruker Ultraflex II MALDI-TOF-TOF equipped with the SMART beam laser. The matrix-assisted laser desorption/ionization (MALDI) was operated in positive ion, reflector mode. Ions were generated by a pulsed beam laser (66.7 Hz) and accelerated to a kinetic energy of 25 kV. External calibration of the MALDI-TOF was achieved by using the monoisotopic peaks of a peptide calibration mixture containing angiotensin II (*m/z *1046.542), bombesin (*m/z *1629.823), ACTH (*m/z *2465.199), and somatostatin (*m/z *3147.472). Calibration of the instrument was performed before each run.

### Peptide synthesis

The peptide GVGGPGHKARVLAEAMSQVTNPATI spanning the CA-SP1 junction was made by the University of Oklahoma Health Sciences Center Molecular Biology-Proteomics Facility. The peptide was obtained by automated solid phase synthesis using Fmoc chemistry and purified by reverse phase HPLC. The peptide was analyzed by mass spectrometry and confirmed to have purity greater than 98%.

### Construction of hypothetical structures

Since the crystal structure of bevirimat was not available, BVM and its C-28 analogs were modeled using PyMOL Molecular Graphics System (DeLano Scientific LLC 2008). The hypothetical helical structures in Figure 6 were constructed in Discovery Studio Client v2.5.0.9164 using six identical helices. Only the 24 residue sequence spanning the helix seen in NMR experiments [34] (GHKARVLAEAMSQVTNSATIMMQR, PDB ID 1U57) was used. The rotational orientation of individual helices was manually constructed and bevirimat was manually docked into the bundle in accordance with crosslinking data. The complex was energy-optimized using the Standard Dynamic Cascade protocol. The hypothetical structure shown in Additional File 1: Additional Figure 2 was created in PyMOL by merging the common 9 amino acid sequence (LEEMMTACQ) of the C-terminal domain of CA [29,30] (PDB ID 1AUM) with that of the NMR structure of the CA-SP1 region [34] (PDB ID 1U57).

## Competing interests

The authors declare that they have no competing interests.

## Authors' contributions

ATN carried out the experimental work and wrote the paper. CLF and KWJ helped with the mass spectrometry experiments and interpretation. TJN and KS provided background information and interpretative input, GMA assisted with structural interpretation and editing of the manuscript, MS conceived the study, oversaw its execution and edited the manuscript. All authors read and approved the final manuscript.

## Supplementary Material

Additional file 1 **Additional Table 1 - Detail of mass analysis of all peptide fragments used to generate the peptide map for HIV-1 Gag**. Additional Table 2 - Detail of mass analysis of all non-crosslinked and crosslinked peptide fragments after protease digestion of crosslinked C-28/Gag. Additional Table 3 - Detail of mass analysis of all non-crosslinked and crosslinked peptide fragments after protease digestion of crosslinked C-30/Gag. Additional Table 4 - Detail of mass analysis of all non-crosslinked and crosslinked peptide fragments after protease digestion of crosslinked C-28 and mutants Gag. Additional Table 5 - Detail of mass analysis of C-28 crosslinked MHR (residues 291-302) of mutants HIV-1 Gag. Additional Table 6 - Contacts between drug and peptide in model A and model B helices. Additional Figure 1 - Detail of mass spectra showing ion masses corresponding to the predicted masses of analog-peptide adducts derived from mutant particles. Additional Figure 2 - Illustrative cartoon of fused crystallographic structure of CA C-terminal domain (PDB ID: 1AUM) and NMR structure of SP1 (PDB ID: 1U57). Click here for file
