# Supplementary material for: The prototype HIV-1 maturation inhibitor, bevirimat, binds to the CA-SP1 cleavage site in immature Gag particles
Source: Retrovirology. 2011 Dec 7;8:101. doi: 10.1186/1742-4690-8-101 (PMC3267693; doi:10.1186/1742-4690-8-101)
Supplement: Additional file 1 — Additional Table 1 - Detail of mass analysis of all peptide fragments used to generate the peptide map for HIV-1 Gag. Additional Table 2 - Detail of mass analysis of all non-crosslinked and crosslinked peptide fragments after protease digestion of crosslinked C-28/Gag. Additional Table 3 - Detail of mass analysis of all non-crosslinked and crosslinked peptide fragments after protease digestion of crosslinked C-30/Gag. Additional Table 4 - Detail of mass analysis of all non-crosslinked and crosslinked peptide fragments after protease digestion of crosslinked C-28 and mutants Gag. Additional Table 5 - Detail of mass analysis of C-28 crosslinked MHR (residues 291-302) of mutants HIV-1 Gag. Additional Table 6 - Contacts between drug and peptide in model A and model B helices. Additional Figure 1 - Detail of mass spectra showing ion masses corresponding to the predicted masses of analog-peptide adducts derived from mutant particles. Additional Figure 2 - Illustrative cartoon of fused crystallographic structure of CA C-terminal domain (PDB ID: 1AUM) and NMR structure of SP1 (PDB ID: 1U57). [file 1742-4690-8-101-S1.PDF]

Additional file for Nguyen et al. The prototype HIV-1 maturation inhibitor, bevirimat, binds to the CA-SP1 cleavage site in immature Gag particles

Additional Table 1 - Detail of mass analysis of all peptide fragments used to generate the peptide map for HIV-1 Gag.

Peptide Fragment #	Theoretical monoisotopic masses(+1) m/z	Observed monoisotopic masses (+1) m/z	% difference from theoretical monoisotopic mass	Signal/Noise	Intensity	Area
Lys-C digestion						
104-110	889.43	No				
19-26	896.58	No				
404-410	901.48	No				
381-388	1019.55	1019.46	-0.0084	321.9	95678	17997
262-272	1112.69	1112.58	-0.0102	309.7	168436	30692
303-314	1359.72	1359.66	-0.0044	191.2	86180	18225
425-436	1438.67	1438.62	-0.0038	829.5	199752	38721
1--15	1569.97	1570.03	0.0038	97	22176	5100
291-302	1634.8	1634.90	0.0058	231.3	99081	25429
315-331	2033.94	2034.01	0.0036	14.3	6716	2721
482-500	2067.04	2067.07	0.0015	776.3	340134	1E+05
273-290	2074.15	2074.38	0.0108	106.1	32945	12137
360-380	2272.22	2271.99	-0.0103	443.3	178895	62572
336-359	2369.11	2369.23	0.0052	53.2	25074	7565
443-475	3674.78	3674.98	0.0056	355.5	125770	62012
163-202	4233.05	4232.92	-0.0031	48.1	24017	14151
115-157	4676.34	4676.45	0.0023	205.4	68793	40757
203-263	6728.31	No				
33-95	7277.77	No				
Glu-C digestion						
313-319	907.43	No				
469-477	1019.5	1019.54	0.0042	69.6	22741	5811
168-177	1093.56	1093.69	0.0121	17.9	6292	2236
420-428	1105.44	1105.61	0.0149	14	4135	1292
43-52	1115.62	1115.74	0.0110	55.9	33455	6549
1--12	1213.77	1213.91	0.0112	500.6	118350	19472
231-245	1532.76	1532.92	0.0102	12.8	4210	1243
246-260	1675.83	1675.99	0.0098	47.2	32360	7622
483-500	1938	1938.13	0.0066	47.4	13740	3907
56-73	1971.99	1971.81	-0.0091	13.9	5969	2795
346-365	2070	2070.05	0.0025	11.8	8496	2966
292-307	2076.07	2076.13	0.0027	10.4	4348	2586
212-230	2167.1	2167.17	0.0031	8.6	4381	3369
399-419	2541.29	2541.22	-0.0026	11.8	3303	1559
320-344	2637.43	2637.34	-0.0035	20.6	10532	4985
178-203	2756.32	2756.23	-0.0034	37.6	19170	10084
18-40	2791.67	No				
429-454	3020.61	3020.57	-0.0015	5.5	2102	1207
75-99	3034.61	3034.76	0.0049	23.3	7655	3547
261-291	3701.11	No				
366-398	3856.92	3856.89	-0.0008	5.7	2859	2252
108-160	5845.03	No				
Arg-C digestion						
287-294	958.51	958.49	-0.0022	19.3	16884	8277
491-500	1024.46	1024.58	0.0114	44.9	32899	28533
276-286	1295.67	1295.65	-0.0013	52.6	24073	12754
265-272	1324.85	1324.94	0.0070	30.9	21098	12921
215-229	1580.85	1580.88	0.0021	22.5	13324	9845
44-58	1649.8	1649.70	-0.0062	38.6	33850	19220
5--20	1787.97	1788.11	0.0080	36.3	25684	21048
77-91	1836.94	1836.82	-0.0068	28.6	28717	19864
59-76	1997.07	1997.18	0.0057	33.3	15322	9354
21-39	2265.35	2265.30	-0.0024	15.9	24081	16700
388-406	2307.13	2307.10	-0.0013	14.8	13267	8833
362-384	2519.31	2519.16	-0.0060	15.3	13722	9687
410-429	2523.15	No				
465-490	2977.52	2977.48	-0.0015	28.1	16943	11681
233-264	3496.75	3496.84	0.0025	6.2	1964	1283
430-464	3976.04	No				
306-361	6036.95	No				
92-150	6561.34	No				
151-214	7073.45	No				

Additional Table 2 - Detail of mass analysis of all non-crosslinked and crosslinked peptide fragments after protease digestion of crosslinked C-28/Gag.

Peptide Fragment #	Theoretical monoisotopic masses(+1) m/z	Observed monoisotopic masses (+1) m/z	% difference from theoretical monoisotopic mass	Signal/Noise	Intensity	Area	Theoretical monoisotopic masses+BVM (+1) m/z	Crosslinked observed ?
Lys-C digestion								
104-110	889.43	No					1730.93	No
19-26	896.58	No					1738.08	No
404-410	901.48	901.42	-0.0068	17.1	4730	973	1742.98	No
381-388	1019.55	1019.50	-0.0050	225	62060	8787	1861.05	No
262-272	1112.69	1112.84	0.0135	122.1	43516	6015	1954.19	No
303-314	1359.72	1359.66	-0.0044	30.1	12171	2077	2201.22	No
425-436	1438.67	1438.62	-0.0038	74.2	30030	5128	2280.17	No
1--15	1569.97	1570.19	0.0142	161.1	58666	11762	2411.47	No
291-302	1634.80	1634.75	-0.0032	106.6	49021	10266	2476.30	2476.57
315-331	2033.94	2034.13	0.0092	528.9	254285	65592	2875.44	No
482-500	2067.04	2066.97	-0.0032	891.7	385558	110773	2908.54	No
273-290	2074.15	2074.30	0.0073	326.8	169973	43599	2915.65	No
360-380	2272.22	2272.12	-0.0044	25	11497	4815	3113.72	No
336-359	2369.11	2369.36	0.0107	30.9	11008	3288	3210.61	3210.84
443-475	3674.78	3674.71	-0.0019	288.6	116867	41494	4516.28	No
163-202	4233.05	4233.39	0.0081	159	63564	31149	5074.55	No
115-157	4676.34	4676.56	0.0046	471.2	223131	105872	5517.84	No
203-263	6728.31	No					7569.81	No
33-95	7277.77	No					8119.27	No
Glu-C digestion								
313-319	907.43	No					1748.93	No
469-477	1019.50	1019.49	-0.0006	44.2	12730	2989	1861.00	No
168-177	1093.56	1093.69	0.0115	20.2	6841	1615	1935.06	No
420-428	1105.44	1105.58	0.0122	17.4	5891	1761	1946.94	No
43-52	1115.62	1115.56	-0.0050	22.3	6426	1928	1957.12	No
1--12	1213.77	1213.88	0.0090	1122.1	230197	38308	2055.27	No
231-245	1532.76	1532.97	0.0136	16.3	6434	3366	2374.26	No
246-260	1675.83	1675.99	0.0096	13.7	3028	1416	2517.33	No
483-500	1938.00	1938.09	0.0047	130.8	47694	11419	2779.50	No
56-73	1971.99	1971.88	-0.0058	9.8	2719	1010	2813.49	No
346-365	2070.00	2069.90	-0.0048	30.7	9452	2700	2911.50	No
292-307	2076.07	2076.02	-0.0026	11.2	7074	2551	2917.57	2917.35
212-230	2167.10	2167.33	0.0108	7.8	3134	1484	3008.60	No
399-419	2541.29	2541.25	-0.0016	8.4	2856	1126	3382.79	No
320-344	2637.43	2637.60	0.0065	9	4500	3514	3478.93	No
178-203	2756.32	2756.11	-0.0076	16.5	8104	2877	3597.82	No
18-40	2791.67	2791.78	0.0038	7.7	3195	1499	3633.17	No
429-454	3020.61	No					3862.11	No
75-99	3034.61	3034.54	-0.0022	10.1	3746	2399	3876.11	No
261-291	3701.11	No					4542.61	No
366-398	3856.92	3857.11	0.0049	14	5467	2581	4698.42	No
108-160	5845.03	No					6686.53	No
Arg-C digestion								
295-299	667.31	No					1508.81	1508.89
287-294	958.51	958.62	0.0113	12.9	10259	3014	1800.01	No
491-500	1024.46	1024.36	-0.0101	6.9	5036	1698	1865.96	No
276-286	1295.67	1295.79	0.0090	8.4	6088	2541	2137.17	No
265-272	1324.85	1324.98	0.0098	16.1	11375	3671	2166.35	No
215-229	1580.85	1581.06	0.0130	173.1	92143	20825	2422.35	No
44-58	1649.80	1649.91	0.0067	13.9	9814	3217	2491.30	No
5--20	1787.97	1788.24	0.0151	14.1	10325	5991	2629.47	No
77-91	1836.94	1837.03	0.0050	345.3	191106	45878	2678.44	No
59-76	1997.07	1997.19	0.0062	8	4628	3989	2838.57	No
21-39	2265.35	2265.29	-0.0028	10.5	6065	4468	3106.85	No
388-406	2307.13	2307.25	0.0052	430.6	213847	62913	3148.63	No
362-384	2519.31	2519.39	0.0032	481.6	349952	125499	3360.81	No
410-429	2523.15	2523.24	0.0037	123.1	64118	20856	3364.65	No
465-490	2977.52	2977.64	0.0040	511	371291	152556	3819.02	No
233-264	3496.75	3497.02	0.0078	25	16604	6651	4338.25	No
430-464	3976.04	3976.43	0.0098	22.5	15663	7647	4817.54	No
306-361	6036.95	No					6878.45	No
92-150	6561.34	No					7402.84	No
151-214	7073.45	No					7914.95	No

Additional Table 3 - Detail of mass analysis of all non-crosslinked and crosslinked peptide fragments after protease digestion of crosslinked C-30/Gag.

Peptide Fragment #	Theoretical monoisotopic masses(+1) m/z	Observed monoisotopic masses (+1) m/z	% difference from theoretical monoisotopic mass	Signal/Noise	Intensity	Area	Theoretical monoisotopic masses+BVM (+1) m/z	Crosslinked observed ?
Lys-C digestion								
104-110	889.43	No					1569.91	No
19-26	896.58	896.48	-0.0113	23.4	22371	4311	1577.06	No
404-410	901.48	901.47	-0.0008	72.1	66226	36692	1581.96	No
381-388	1019.55	1019.49	-0.0064	74.5	53446	9828	1700.03	No
262-272	1112.69	1112.68	-0.0012	60.3	100205	26747	1793.17	No
303-314	1359.72	1359.74	0.0016	175.3	167873	53205	2040.20	No
425-436	1438.67	1438.77	0.0067	43.8	45580	18056	2119.15	No
1--15	1569.97	1569.88	-0.0057	130.3	149428	39365	2250.45	No
291-302	1634.8	1635.02	0.0135	86.6	104301	35412	2315.28	No
315-331	2033.94	2033.85	-0.0043	58.2	109809	35655	2714.42	No
482-500	2067.04	2067.24	0.0096	583	784689	395781	2747.52	No
273-290	2074.15	No					2754.63	No
360-380	2272.22	2272.19	-0.0011	54.1	73503	34982	2952.70	No
336-359	2369.11	2369.06	-0.0022	87.9	145925	59304	3049.59	No
443-475	3674.78	3675.20	0.0113	52.7	65147	31567	4355.26	No
163-202	4233.05	4233.28	0.0055	131.5	224823	141430	4913.53	No
115-157	4676.34	4676.75	0.0089	20.1	28349	15987	5356.82	No
203-263	6728.31	No					7408.79	No
33-95	7277.77	No					7958.25	No
Glu-C digestion								
313-319	907.43	No					1587.91	No
469-477	1019.5	1019.60	0.0096	30.1	11132	2363	1699.98	No
168-177	1093.56	1093.58	0.0018	37	6476	2958	1774.04	No
420-428	1105.44	1105.50	0.0054	40.5	11627	4805	1785.92	No
43-52	1115.62	1115.57	-0.0049	77.7	42709	9947	1796.10	No
1--12	1213.77	1213.86	0.0075	11.8	5722	2312	1894.25	No
231-245	1532.76	1532.72	-0.0024	30.8	16050	10840	2213.24	No
246-260	1675.83	1676.03	0.0119	19.5	5798	3325	2356.31	No
483-500	1938	1937.93	-0.0038	35.1	11385	3408	2618.48	No
56-73	1971.99	1972.11	0.0062	13.9	5785	2353	2652.47	No
346-365	2070	2070.06	0.0030	13.7	7253	3236	2750.48	2750.60
292-307	2076.07	2076.23	0.0075	11.9	4565	2044	2756.55	No
212-230	2167.1	2167.34	0.0108	19.7	14188	13334	2847.58	No
399-419	2541.29	2541.18	-0.0043	12.2	10101	7516	3221.77	No
320-344	2637.43	2637.56	0.0049	42.7	14356	4603	3317.91	No
178-203	2756.32	No					3436.80	No
18-40	2791.67	2791.51	-0.0059	8.5	3754	1489	3472.15	No
429-454	3020.61	No					3701.09	No
75-99	3034.61	3034.50	-0.0037	11.4	3895	1634	3715.09	No
261-291	3701.11	No					4381.59	No
366-398	3856.92	3857.04	0.0032	27.3	9443	4238	4537.40	No
108-160	5845.03	No					6525.51	No
Arg-C digestion								
287-294	958.51	958.47	-0.0041	17.9	24861	12611	1638.99	No
491-500	1024.46	1024.52	0.0061	15	20833	17265	1704.94	No
276-286	1295.67	1295.76	0.0069	26.6	27256	18644	1976.15	No
265-272	1324.85	1325.02	0.0130	49.4	39480	20288	2005.33	No
215-229	1580.85	1580.99	0.0089	16.5	25917	18844	2261.33	No
44-58	1649.8	1649.77	-0.0020	26.6	39510	22481	2330.28	No
5--20	1787.97	1787.99	0.0013	13.4	19971	13791	2468.45	No
77-91	1836.94	1836.89	-0.0028	34.4	14414	9371	2517.42	No
59-76	1997.07	1997.22	0.0074	21.7	17928	13842	2677.55	No
21-39	2265.35	2265.27	-0.0035	13.3	5264	3730	2945.83	No
388-406	2307.13	2307.31	0.0078	11.4	5019	4277	2987.61	No
362-384	2519.31	2519.39	0.0031	9.6	9193	6288	3199.79	3199.66
410-429	2523.15	2523.40	0.0098	10.6	8864	7497	3203.63	No
465-490	2977.52	2977.66	0.0046	20.7	8303	7639	3658.00	No
233-264	3496.75	3497.18	0.0123	8.8	7914	6290	4177.23	No
430-464	3976.04	No					4656.52	No
306-361	6036.95	No					6717.43	No
92-150	6561.34	No					7241.82	No
151-214	7073.45	No					7753.93	No

Additional Table 4 - Detail of mass analysis of all non-crosslinked and crosslinked peptide fragments after protease digestion of crosslinked C-28 and mutants Gag.

MUTANT L231M Peptide Fragment #	Theoretical monoisotopic masses(+1) m/z	Observed monoisotopic masses (+1) m/z	% difference from theoretical monoisotopic mass	Signal/Noise	Intensity	Area	Theoretical monoisotopic masses+BVM (+1) m/z	Crosslinked observed ?
Lys-C digestion								
104-110	889.43	889.47	0.0047	25.1	10530	3607	1730.93	No
19-26	896.58	No					1738.08	No
404-410	901.48	901.43	-0.0053	45.1	19661	5177	1742.98	No
381-388	1019.55	1019.60	0.0048	62.9	21470	5468	1861.05	No
262-272	1112.69	1112.67	-0.0017	97.1	42580	8513	1954.19	No
303-314	1359.72	1359.69	-0.0024	157.1	39004	8071	2201.22	No
425-436	1438.67	1438.71	0.0026	41.3	19250	6322	2280.17	No
1--15	1569.97	1570.07	0.0061	23.6	6826	3596	2411.47	No
291-302	1634.80	1635.03	0.0141	105.0	68052	16622	2476.30	2476.40
315-331	2033.94	2033.93	-0.0004	14.4	7413	5691	2875.44	No
482-500	2067.04	2067.00	-0.0020	63.1	23311	9406	2908.54	No
273-290	2074.15	2074.15	0.0000	12.0	8084	5933	2915.65	No
360-380	2290.17	2290.07	-0.0046	31.3	16055	7165	3131.67	No
336-359	2369.11	2369.05	-0.0024	21.7	9521	3656	3210.61	No
443-475	3674.78	3674.72	-0.0016	9.7	4744	3795	4516.28	No
163-202	4233.05	4233.42	0.0088	6.6	1904	1247	5074.55	No
115-157	4676.34	4676.76	0.0091	11.8	5384	2853	5517.84	No
203-263	6728.31	No					7569.81	No
33-95	7277.77	No					8119.27	No
**A1V**								
104-110	889.43	889.51	0.0091	68.4	48335	8215	1730.93	No
19-26	896.58	No					1738.08	No
404-410	901.48	No					1742.98	No
381-388	1019.55	1019.63	0.0082	32.7	20271	4643	1861.05	No
262-272	1112.69	1112.85	0.0147	33.4	18840	4310	1954.19	No
303-314	1359.72	1359.87	0.0113	27.5	7752	3074	2201.22	No
425-436	1438.67	1438.79	0.0081	20.4	7131	2703	2280.17	No
1--15	1569.97	1569.93	-0.0024	19.6	7796	3873	2411.47	No
291-302	1634.80	1634.86	0.0038	42.8	34988	10017	2476.30	2476.19
315-331	2033.94	2033.93	-0.0003	19.1	8452	3681	2875.44	No
482-500	2067.04	2067.07	0.0013	55.6	23460	8364	2908.54	No
273-290	2074.15	2074.40	0.0119	22.6	14021	7454	2915.65	No
360-380	2300.24	No					3141.74	No
336-359	2369.11	2369.26	0.0062	17.0	13221	6217	3210.61	No
443-475	3674.78	3674.99	0.0058	66.8	54554	23656	4516.28	No
163-202	4233.05	No					5074.55	No
115-157	4676.34	4676.77	0.0091	6.0	2668	1865	5517.84	No
203-263	6728.31	No					7569.81	No
33-95	7277.77	No					8119.27	No

... continued

Additional Table 4 (continued)

Δ14M

104-110	889.43	889.43	0.0001	46.9	21385	7178	1730.93	No
19-26	896.58	896.52	-0.0072	191.2	83197	21257	1738.08	No
403-409	901.48	901.63	0.0168	80.6	37265	15809	1742.98	No
380-387	1019.55	1019.66	0.0112	44.1	22093	5827	1861.05	No
262-272	1112.69	1112.69	0.0004	17.4	5219	1044	1954.19	No
303-314	1359.72	1359.88	0.0121	23.0	10763	4097	2201.22	No
424-435	1438.67	No					2280.17	No
1--15	1569.97	1570.01	0.0027	19.8	13253	5689	2411.47	No
291-302	1634.80	1634.90	0.0061	28.3	13016	3151	2476.30	2476.22
315-331	2033.94	No					2875.44	No
481-499	2067.04	2067.12	0.0037	78.6	29005	9094	2908.54	No
273-290	2074.15	2074.32	0.0083	12.5	4690	1827	2915.65	No
360-379	2141.17	2141.25	0.0039	17.1	6311	3466	2982.67	No
336-359	2369.11	2369.40	0.0123	15.5	7256	3863	3210.61	No
442-474	3674.78	3674.99	0.0058	10.8	4557	2378	4516.28	No
163-202	4233.05	No					5074.55	No
115-157	4676.34	No					5517.84	No
203-263	6728.31	No					7569.81	No
33-95	7277.77	No					8119.27	No

A3V

104-110	889.43	889.46	0.0028	31.8	15963	4880	1730.93	No
19-26	896.58	No					1738.08	No
404-410	901.48	No					1742.98	No
381-388	1019.55	1019.62	0.0064	107.9	33703	8431	1861.05	No
262-272	1112.69	1112.69	0.0004	220.8	90861	20857	1954.19	No
303-314	1359.72	1359.78	0.0040	23.2	19622	8967	2201.22	No
425-436	1438.67	1438.86	0.0133	16.6	17545	10232	2280.17	No
1--15	1569.97	1569.91	-0.0039	17.2	15841	12408	2411.47	No
291-302	1634.80	1634.76	-0.0027	35.8	14805	6687	2476.30	No
315-331	2033.94	2033.86	-0.0041	101.0	48980	20859	2875.44	No
482-500	2067.04	2067.04	-0.0002	635.8	373990	136819	2908.54	No
273-290	2074.15	2074.22	0.0035	17.7	8714	5844	2915.65	No
360-380	2300.24	2300.08	-0.0070	17.0	13422	10285	3141.74	No
336-359	2369.11	2369.14	0.0012	32.7	23336	12884	3210.61	No
443-475	3674.78	No					4516.28	No
163-202	4233.05	No					5074.55	No
115-157	4676.34	No					5517.84	No
203-263	6728.31	No					7569.81	No
33-95	7277.77	No					8119.27	No

H226Y

104-110	889.43	No					1730.93	No
19-26	896.58	No					1738.08	No
404-410	901.48	No					1742.98	No
381-388	1019.55	1019.61	0.0061	17.6	27893	14372	1861.05	No
262-272	1112.69	1112.70	0.0005	48.3	35913	18210	1954.19	No
303-314	1359.72	1359.78	0.0046	16.7	11123	5232	2201.22	No
425-436	1438.67	1438.82	0.0101	17.0	10804	4756	2280.17	No
1--15	1569.97	1569.94	-0.0018	17.4	27552	22348	2411.47	No
291-302	1634.80	1635.01	0.0128	30.8	25438	20741	2476.30	2476.32
315-331	2033.94	2034.06	0.0058	11.7	7691	6158	2875.44	No
482-500	2067.04	2066.96	-0.0040	26.1	20942	11631	2908.54	No
273-290	2074.15	2074.19	0.0018	25.5	17423	9775	2915.65	No
360-380	2272.22	No					3113.72	No
336-359	2395.11	2395.36	0.0103	25.6	24227	17590	3236.61	No
443-475	3674.78	No					4516.28	No
163-202	4233.05	No					5074.55	No
115-157	4676.34	No					5517.84	No
203-263	6728.31	No					7569.81	No
33-95	7277.77	No					8119.27	No

Additional Table 5 - Detail of mass analysis of C-28 crosslinked MHR (residues 291-302) of mutants HIV-1 Gag.

Mutant	Theoretical mass + BVM (+1)	Observed mass (+1)	% difference from theoretical mass	Signal/Noise	Intensity	Area
L363M	2476.30	2476.40	0.0042	8.9	4199	2507
A364V	2476.30	2476.19	-0.0043	9.0	3625	2694
ΔM377	2476.30	2476.22	-0.0023	19.5	15303	10742
H358Y	2476.30	2476.32	0.0003	35.8	37103	29888

Additional Table 6 - Contacts between drug and peptide in model A and model B helices.
Contacts within 3.7 Å are listed along with hydrogen bonds identified by Swiss PDB Viewer using default settings for distance and angle.
Amino acids are numbered by their position in Gag. The peptide sequence used for modeling the helices encompassed amino acids 357-380 and the starting model fused information from crystal and NMR structures as described in Materials and Methods and Supplemental Figure 2.

	Model A		Model B	
	AAs in contact	Polar contacts	AAs in contact	Polar contacts
Chain A	Gly 357		Ala 360	
	Arg 361		Leu 363	
	Ala 364		Met 367	
	Ser 368	OG to CHE1/002 2.6 Å	Thr 371	OG to CHE1/003 2.6 Å
	Thr 371			
Chain B	Gly 357		His 358	
	Ala 360		Arg 361	
	Arg 361	NE to CPE 1/001 2.8 Å NH2 to CPE 1/001 2.9 Å	Glu 365	
	Ala 364			
	Ser 368	OG to CHE 1/003 3.4 Å		

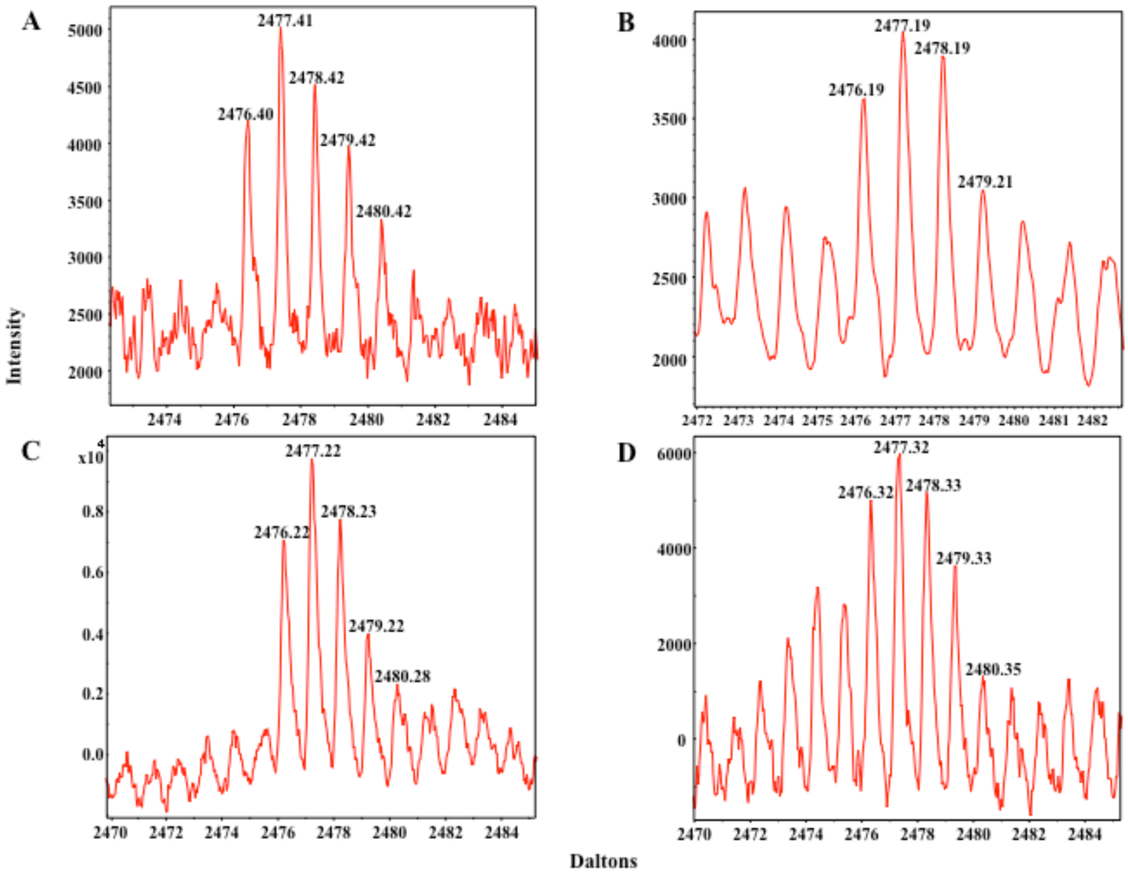

Additional Figure 1 - Detail of mass spectra showing ion masses corresponding to the predicted masses of analog-peptide adducts derived from mutant particles.

(A) Lys-C peptide, L363M mutant Gag residues 291-302, linked to the C-28 analog. (B) Lys-C peptide, A364V mutant Gag residues 291-302, linked to the C-28 analog. (C) Lys-C peptide, ΔM377 mutant Gag residues 291-302, linked to the C-28 analog. (D) Lys-C peptide, H358Y mutant Gag residues 291-302, linked to the C-28 analog.

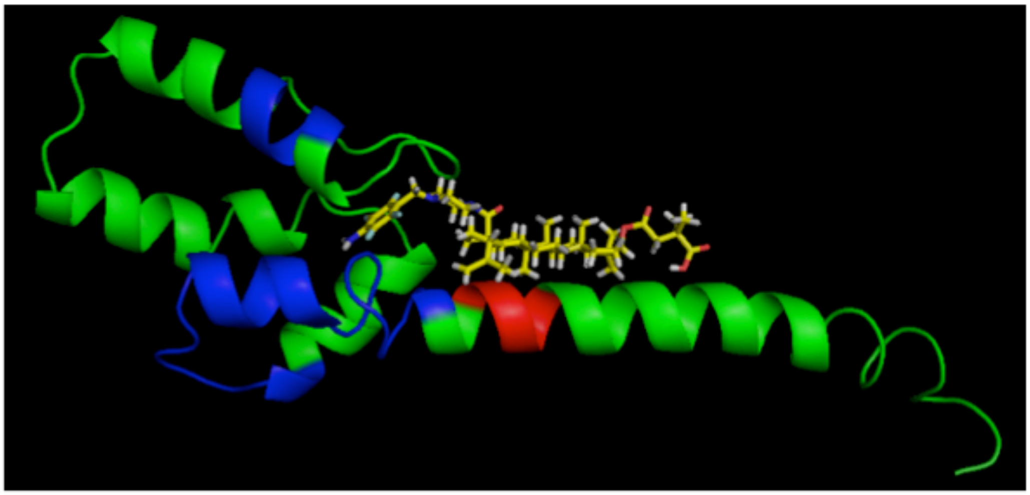

Additional Figure 2 - **Illustrative cartoon of fused crystallographic structure of CA C-terminal domain (PDB ID: 1AUM) and NMR structure of SP1 (PDB ID: 1U57)**. The common regions of peptides found linked to the C-28 analog are indicated in blue and the common region of peptides found linked to the C-30 is indicated in red. The C-28 analog is shown positioned along the SP1 helix with the photo-reactive group oriented in the direction of two identified linking sites.
